# The Severity of Oxidative Stress in Comorbid Chronic Obstructive Pulmonary Disease (COPD) and Hypertension: Does it Depend On ACE and AGT Gene Polymorphisms?

**DOI:** 10.25122/jml-2019-0108

**Published:** 2019

**Authors:** Mariya Marushchak, Khrystyna Maksiv, Inna Krynytska, Olha Dutchak, Nina Behosh

**Affiliations:** 1. Department of Functional and Laboratory Diagnostics, I. Horbachevsky Ternopil National Medical University, Ternopil, Ukraine; 2. Department of Medical Rehabilitation, I. Horbachevsky Ternopil National Medical University, Ternopil, Ukraine; 3. Department of Pediatrics, Institute of Postgraduate Education, I. Horbachevsky Ternopil National Medical University,Ternopil, Ukraine

**Keywords:** COPD, hypertension, oxidative stress, gene polymorphism, ACE - Angiotensin-Converting Enzyme, AGT - Angiotensinogen, COPD - Chronic Obstructive Pulmonary Disease, H2O2 - Hydrogen Peroxide, O2• - Superoxide, ROS - Reactive Oxygen Species

## Abstract

There is an increasing number of studies suggesting the role of genetic factors in the development and progression of chronic obstructive pulmonary disease and hypertension. Therefore, our study aimed to establish the role of ACE and AGT gene polymorphisms in the mechanisms behind the development of oxidative stress in patients with concomitant chronic obstructive pulmonary disease and hypertension. The study group consisted of 96 patients: Group 1 (individuals with a chronic obstructive pulmonary disease), Group 2 (individuals with arterial hypertension), Group 3 (individuals with a chronic obstructive pulmonary disease and arterial hypertension). The control group consisted of 20 healthy subjects. ACE and AGT gene polymorphisms were determined by polymerase chain reaction amplification. Detection of intracellular reactive oxygen species levels was performed by EPICS XL cytometer (Beckman Coulter, USA) with dichlorodihydrofluorescein diacetate and dihydroethidium. Serum levels of 8-isoprostane were assayed with ELISA, Cayman Chemicals (USA). No significant correlations between ACE and AGT gene polymorphisms and parameters of oxidative stress in a setting of comorbid chronic obstructive pulmonary disease and hypertension were observed. However, the increase in oxidative stress parameters was observed to be the most significant in patients with chronic obstructive pulmonary disease + hypertension and with I/I genotype of the ACE gene, which was due to their lowest values in virtually healthy individuals. This suggests that I/I genotype may be associated with lower levels of reactive oxygen species production compared with other genotypes.

## Introduction

Globally, chronic obstructive pulmonary disease (COPD) is the most common chronic respiratory condition [1-3]. More than 384 million cases of COPD were registered worldwide in 2010 [4]. COPD is the third most frequent cause of death in the United States, preceded by cardiovascular disease and cancer, causing up to $50 billion in direct and indirect health care costs [1]. In the countries of Europe, the prevalence of COPD ranges from 4% to 10% [5]. However, of the 50 nation-states of Europe, only 19 (38%) have robust COPD statistics [6]. The values of mortality per 100.000 of the population are within a wide range, from 95 (Kyrgyzstan) to 6 (Greece). In Ukraine, COPD-associated mortality is much higher than in other European states, i.e., over 80 [7].

• COPD often has various concomitant conditions, which are generally more prevalent in patients with COPD than the overall population. A study of COPD-associated comorbidities conducted by Soriano JB et al. has demonstrated that COPD patients were at increased risk for pneumonia (a relative risk ratio of 16.00), osteoporosis (3.14), myocardial infarction (1.75), angina (1.67), bone fracture (1.58), and glaucoma (1.29) [8]. Cardiovascular disease, especially hypertension, is often concomitant with COPD and contributes to a heightened risk of mortality [4]. Ukrainian statistics report concomitant hypertension in 35% of COPD patients; COPD combined with concomitant coronary artery disease is responsible for 62% of morbidity in older COPD patients [9].
• Pathogenetic pathways of either hypertension or COPD are both dependent on oxidative stress [10]. The latter constitutes a failure of anti-oxidation defense systems to keep reactive oxygen species (ROS) and reactive nitrogen species in check [11,12]. There are multiple sources of ROS within the cell, i.e., mitochondria, xanthine oxidase, NO-synthase (in an uncoupled form), and NAD(P)H oxidase. Consistent elevations of mitochondrial ROS outputs trigger a devastating cascade of damage to mitochondrial DNA; any further compromises of mitochondrial function contribute to an increase in ROS and cell damage [13]. One recent study suggested that mitochondrial ROS were signaling molecules that launched the release of pro-inflammatory cytokines [14]; a dysregulation of this response plays an essential role in the development of inflammation [15].

Although there is research-based evidence demonstrating the involvement of ROS in atherosclerosis and their stimulatory contribution to inflammation [16], there is hardly any direct evidence to show that the increased oxidative stress of COPD increases cardiovascular risks [17]. Besides, ROSs are involved in modified vascular responses, dysfunction of endothelium, and vascular remodeling often seen in patients with COPD. The latter changes include cellular proliferation in the vascular wall and the constriction of blood vessels [18]. Along with that, critical pathogenetic constituents of hypertension include lower antioxidant enzymatic activity, ROS-mediated attenuation of endothelial nitric oxide (NO) and endothelial dysfunction, the latter resulting in clinically manifest vasoconstriction [19]. In our previous study [20], we have demonstrated that ROS production increases within the cells; the severity of this finding correlated with the degree of bronchial obstruction in patients with COPD and concomitant hypertension.

There is an increasing number of studies suggesting the role of genetic factors in the development and progression of COPD [21-23] and hypertension [24]. The majority of studies on the genetic aspects of hypertension or COPD have hitherto focused on only one condition. However, the studies of gene polymorphism are of particular importance in comorbid conditions, given the existing similarities between pathogenetic molecular mechanisms of COPD and hypertension. Therefore, our study aimed to establish the role of polymorphism in the angiotensin-converting enzyme (ACE) and angiotensinogen (AGT) genes in the mechanisms behind the development of oxidative stress in patients with concomitant COPD and hypertension.

## Material and Methods

### Patients

This study enrolled 73 in-patients at the University Hospital in Ternopil, Ukraine. Study subjects were randomized into the following three groups: Group 1 (25 COPD-only subjects), Group 2 (28 subjects with COPD and hypertension) and Group 3 (the control group, which included 20 healthy volunteers). The COPD-only group and COPD + hypertension group were matched in terms of age, gender, and smoking status of the patients.

The eligibility criteria included male gender, age 40 to 60 years, and a verified diagnosis of COPD with or without hypertension. Exclusion criteria were asthma, active pulmonary and extrapulmonary tuberculosis, lung tumors, moderate to severe bronchiectasis, lung fibrosis, sarcoidosis, interstitial pulmonary disease with and without fibrosis and α1-antitrypsin deficiency; evidence of clinically significant gastrointestinal, renal, hepatic, immunological, urogenital, neurological and musculoskeletal conditions; psychiatric conditions, substance with or without alcohol abuse; cutaneous lesions, disorders of sensory organs, endocrine disease (including poorly controlled/decompensated diabetes or thyroid disease), serious blood dyscrasias, uncontrolled hepatic disease; patients with cancer who have not remained disease-free for at least five years, inadequately controlled with or without severe cardiovascular conditions.

Diagnosis of COPD was informed by Order 555 of the MoH of Ukraine as of June 27, 2013, and by the guidelines of the American Thoracic Society and European Respiratory Society (GOLD, 2013). The severity of airway obstruction was assessed using the GOLD classification (2008). COPD with moderate (Stage 2) airway obstruction was diagnosed by characteristic clinical features and restricted airflow. The latter was defined as an FEV1/FVC ratio of less than 0.70, i.e., forced expiratory volume in 1 second (FEV1) to forced vital capacity (FVC), which was 50–79% of the expected values. Spirometry was performed using a Spirolab III unit manufactured by Medical International Research (Italy). The test was usually scheduled in the morning and performed in a properly ventilated room. The patients were advised not to eat for 8 hours before the test and to wear comfortable, unrestrictive clothing for the test. Any inhaled bronchodilators were suspended for 12 hours pre-spirometry.

The diagnosis of stage 1 hypertension was performed using the 2018 ESC/ESH Guidelines for the management of arterial hypertension. Stage 1 hypertension was defined as systolic blood pressure (BP) of 140 to 159 mm Hg and/or diastolic BP of 90 to 99 mm Hg. The presence of left ventricular hypertrophy was assessed with an EKG.

### Testing for I/D polymorphism in the ACE gene and the M235T polymorphism in the AGT gene

Genotyping was performed with 2.7 mL samples of venous blood obtained under sterile conditions into Monovettes with EDTA as an anticoagulant. Subsequently, the samples were frozen to be stored at –20ºС. DNA was extracted for molecular genetic tests; polymerase chain reaction (PCR) and length of restriction fragments were further assessed. A standard salt precipitation technique was used to extract DNA from white blood cells of peripheral blood. I/D polymorphism in the ACE gene and M235T polymorphism in the AGT gene were genotyped using a technique of PCR-based restriction fragment length polymorphism (RFLP). 5’-GATGCGCACAAGGTCCT-GTC-3’ (forward) and 5’-CAGGGTGCTGTCCAC-ACTGG ACCCC-3’ (reverse) primers were used. The products of PCR were then digested using 3 units of Tth111I restriction enzyme (supplied by Fermentas); the fragments were separated using 3% ethidium bromide-containing agarose gel and visualized under UV light. In order to test the reliability of the genotyping procedure, double sampling RFLP-PCR was performed in > 20% of samples. No differences were found. The study included an assessment of I/D polymorphism (AluYa5, rs4646994) in the ACE gene. At the test site, a 288 bp fragment (of intron 16) is either inserted or removed. In the case of I/I and D/D homozygous genotypes, one band was seen at 480 or 192 bp, respectively. As for the heterozygous genotype (I/D), both bands were observed simultaneously [25]. The heterozygous variety (M/T) was suggested by the presence of two fragments (165 bp and 141 bp), while the homozygous type was manifest with just one 141 bp (T/T) band; the subjects without the above mutation had only one band, i.e., 165 pb (M/M) [26].

### Assessment of intracellular ROS levels

The EPICS XL cytometer (BeckmanCoulter, USA) was used to perform the flow cytometry test. This flow cytometer was equipped with a 488 nm/15 mW argon-ion laser. A 610 nm bandpass filter was used to detect ethidium red fluorescence. Doublets and subcellular debris were cleared using electronic gating. The subcellular debris was mostly removed with forward scatter (FSC) threshold technology. A minimal threshold of 3000 events (cells) per sample was used to assess each of the test values [27].

Both DCFH-DA (a hydrogen peroxide (H2O2)-specific assay and DHE (superoxide (O2•)- specific ROS assay) are cell-permeable. Free intracellular H2O2 selectively oxidizes dichlorodihydrofluorescein (DCFH) to form dichlorofluorescein (DCF); the latter binds to the DNA, which is seen as the emission of green fluorescence. DHE is respectively oxidized by free intracellular O2• to form ethidium bromide; after binding to the DNA, the latter emits red fluorescence (31–33). Dichlorodihydrofluorescein diacetate (DCFH-DA) (25 mM) and dihydroethidium (DHE) (1.25 mM; Sigma) were added to a suspension of white blood cells and incubated at 25°C for 40 minutes (DCFH-DA) or 20 minutes (DHE). Aliquots were tested on a flow cytometer. Green fluorescence (dichlorofluorescein - DCF) was tested within the 500 to 530 nm range, while red fluorescence (hydroethidine - HE) was evaluated within the 590 to 700 nm range (excitation at 488 nm and emission at 525 to 625 nm in the FL-2 channel). Assay results were presented as percentages of fluorescent white blood cells [28].

### 8-Isoprostane detection

Serum 8-isoprostane levels were obtained with ELISA (8-Isoprostane ELISA Kit [Kit No. 516351), Cayman Chemicals [USA]). The results of the test were given as pg/mL.

### Data Analysis

Statistical analysis was performed with the STATISTICA 7.0 software. The choice of method for statistical analysis depended on whether the distribution of study variables was normal.

Since the distribution of quantitative characteristics was not normal, the descriptive statistics were given as median (Me), lower quartile (Lq), and upper quartile (Uq).

The Kruskal-Wallis test was used for comparative analysis of quantitative variables in three or more groups; the test was acknowledged as statistically significant at p<0.05. Further pairwise comparisons between the groups were performed with the Mann-Whitney U-test; the Bonferroni adjustment was used to assess statistical significance.

## Results

**Figure 1:**
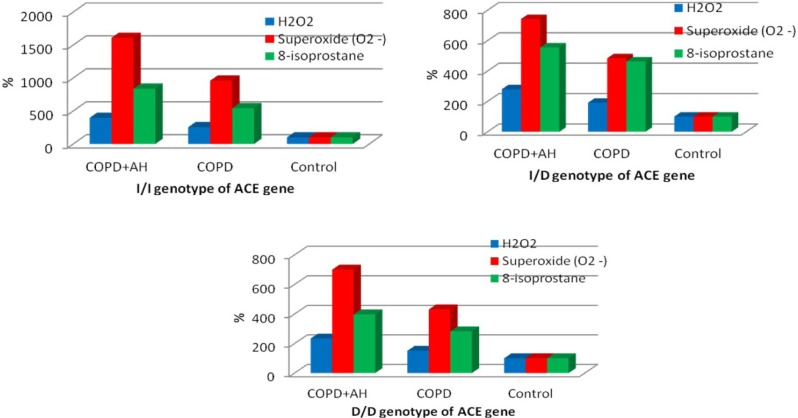
The percentage ratio of changes in oxidative stress parameters within one genotype of the ACE gene.

Among the subjects enrolled in this study, the distribution of polymorphic variants of the ACE gene was as follows: the I/I genotype was found in 28.8% subjects, while I/D and D/D genotypes were found in 52.1% and 19.1% subjects, respectively. The oxidative stress parameters were not found to be significantly dependent on the genotype of the ACE gene in the study groups and the control group, and overall in all study subjects (Table 1). The Kruskal–Wallis test has found significant variances between oxidative stress parameters in patients of different test groups within one genotype of the ACE gene (Table 2). Thus, the patients with I/I, I/D, and D/D genotypes and comorbid COPD and hypertension were found to have the highest values of ROS production and 8-isoprostane levels compared with COPD-only patients and control subjects (Figure 1). It should be emphasized that among the patients with I/I, I/D and D/D genotypes, the test parameters of oxidative stress were significantly higher in patients with comorbid COPD and hypertension when compared with the group of COPD-only patients, a mean 1.5-fold difference. When test parameters of oxidative stress were compared, the most significant increase in RSO and 8-isoprostane levels was found in patients with comorbid COPD+hypertension, and the I/I genotype of the ACE gene (the difference compared with controls). The latter finding is likely attributable to the lowest control values of test parameters found in subjects with I/I genotypes compared with other (I/D and D/D) genotypes of the ACE gene. In this respect, the highest control values and the lowest values in the test groups were found in subjects with the D/D genotype of the ACE gene (Table 1, Figure 1).

**Table 1:** Changes in oxidative stress parameters in COPD-only patients and patients with comorbid COPD and hypertension depending on polymorphisms in the AGT gene (Me [Lq; Uq]).

Parameters	Genotypes			н, р
**In all study subjects (n=73)**				
** **	**I/I (n=21)**	**I/D (n=38)**	**D/D (n=14)**	
**8-isoprostane**	136.00 (87.00, 165.00)	133.00 (47.00, 167.00)	133.00 (95.00, 162.00)	H=0.08 р=0.96
**Н_2_О_2_**	57.10 (45.20, 83.70)	57.00 (35.60, 78.90)	65.90 (44.60, 79.80)	H=0.98 р=0.61
**О2•-**	10.50 (6.40, 15.60)	10.40 (2.90, 14.80)	12.35 (7.40, 14.70)	H=0.65 р=0.72
**COPD+hypertension (n=28)**				
** **	**I/I (n=9)**	**I/D (n=12)**	**D/D (n=7)**	
**8-isoprostane**	176.00 (162.00, 189.00)	172.00 (159.00, 189.50)	155.00 (134.00, 196.00)	H=0.21 р=0.90
**Н_2_О_2_**	83.90 (82.90, 85.40)	83.50 (79.55, 86.95)	79.80 (71.20, 89.60)	H=0.07 р=0.97
**О2•-**	16.10 (15.10, 16.90)	15.95 (15.10, 17.00)	14.70 (13.90, 17.30)	H=0.16 р=0.92
**COPD (n=25)**				
** **	**I/I (n=7)**	**I/D (n=14)**	**D/D (n=4)**	
**8-isoprostane**	114.00 (95.00, 149.00)	143.50 (108.00, 165.00)	110.00 (101.50, 137.00)	H=2.53 р=0.28
**Н_2_О_2_**	54.10 (46.70, 57.10)	57.00 (52.10, 64.20)	51.40 (47.65, 56.40)	H=2.21 р=0.33
**О2•-**	9.60 (7.30, 10.50)	10.40 (8.70, 11.00)	9.05 (8.15, 10.05)	H=2.03 р=0.36
**Control (n=20)**				
	**I/I (n=5)**	**I/D (n=12)**	**D/D (n=3)**	
**8-isoprostane**	21.00 (16.00, 34.00)	31.00 (25.50, 44.00)	39.00 (28.00, 45.00)	H=2.82 р=0.24
**Н_2_О_2_**	21.20 (15.70, 33.40)	29.80 (24.25, 33.70)	34.10 (26.70, 34.90)	H=2.48 р=0.29
**О2•-**	1.00 (0.80, 1.80)	2.15 (1.15, 2.75)	2.10 (1.80, 2.60)	H=3.62 р=0.16

Note: H = Kruskal-Wallis test; p = the significance test of the latter

**Table 2:** Comparative analysis of changes in oxidative stress parameters in COPD-only patients and patients with combined COPD + hypertension within one genotype of the ACE gene.

Parameters	8-isoprostane	Н_2_О_2_	О2•-
**I/I genotype**			
**Kruskal-Wallis test and its respective test of significance**	Н=15.54; р<0.001*	Н=17.45; р<0.001*	Н=17.45; р<0.001*
**Mann–Whitney U test in pairwise comparison of groups**	р1-2=0.005* р2-3=0.003* р1-3=0.004*	р1-2<0.001* р2-3=0.003* р1-3=0.004*	р1-2<0.001* р2-3=0.003* р1-3=0.004*
**I/D genotype**			
**Kruskal-Wallis test and its respective test of significance**	Н=27.08; р<0.001*	Н=32.43; р<0.001*	Н=32.85; р<0.001*
**Mann–Whitney U test in pairwise comparison of groups**	р1-2=0.011* р2-3<0.001* р1-3<0.001*	р1-2<0.001* р2-3<0.001* р1-3<0.001*	р1-2<0.001* р2-3<0.001* р1-3<0.001*
**D/D genotype**			
**Kruskal-Wallis test and its respective test of significance**	Н=8.84; р=0.012*	Н=11.00; р=0.004*	Н=11.00; р=0.004*
**Mann–Whitney U test in pairwise comparison of groups**	р1-2=0.059 р2-3=0.016* р1-3=0.034	р1-2=0.008* р2-3=0.016* р1-3=0.034	р1-2=0.008* р2-3=0.016* р1-3=0.034

Note 1: р1-2 = the significance test when comparing the COPD+hypertension group with the COPD group; р2-3 = the significance test when comparing the COPD + hypertension group with the control group; р1-3 = the significance test when comparing the COPD group with the control group.

Note 2: The level of statistical significance according to Bonferroni adjustment for intergroup comparison is at p<0.017.

Note 3: * = statistically significant results.

**Figure 2:**
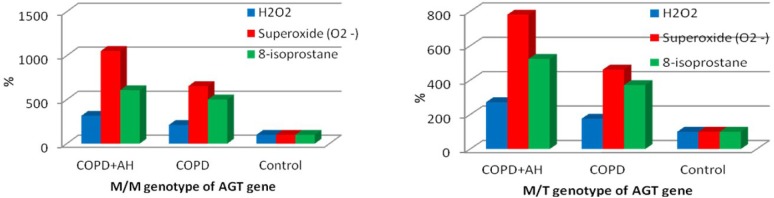
The percentage ratio of changes in oxidative stress parameters within one genotype of the AGT gene.

The polymorphic variants of the AGT gene were distributed as follows: 26.0% subjects had an M/M genotype, 60.3% subjects had an M/T genotype, and 13.7% subjects had a T/T genotype. In test groups, in the control group and overall in study subjects, oxidative stress parameters were not significantly dependent on the genotype of the AGT gene (Table 3). Within the M/M and M/T genotypes of the AGT gene, the Kruskal–Wallis test has found significant variances between oxidative stress parameters in patients of different test groups, while subjects with Т/Т genotype did not have any significant differences regarding the test parameters (Table 4). Thus, patients with M/M and M/T genotypes of the AGT gene and with comorbid COPD and hypertension had the highest test parameters of oxidative stress compared with Group 1 and controls (Figure 2). It should be emphasized that among patients with M/M and M/T genotypes of the AGT gene, significantly higher investigational values of H2O2 and О2•- were observed in patients with comorbid COPD and hypertension compared with the group of COPD-only patients, a mean 1.6-fold difference. That said, there were no statistically significant differences between Group 1 and Group 2 regarding the 8-isoprostane levels in the blood of patients with the M/M genotype of the AGT gene (Table 4). When comparing test parameters of oxidative stress, the most significant increase in oxidative stress parameters was found in COPD+hypertension patients with the M/M genotype of the AGT gene (compared with controls). This was also due to the lower control values of test parameters in subjects with M/M genotype compared with the M/T genotype of the AGT gene (Table 3, Figure 2).

**Table 3:** The key parameters of oxidative stress in groups of COPD-only patients and patients with combined COPD and hypertension depending on polymorphisms of theAGT gene (Me [Lq; Uq]).

Parameters	Genotypes			Н, р
**In all study subjects (n=73)**				
	**M/M (n=19)**	**M/T (n=44)**	**T/T (n=10)**	
**8-isoprostane**	118.00 (35.00, 164.00)	123.50 (49.00, 159.00)	166.50 (155.00, 176.00)	H=4.91 р=0.09
**Н_2_О_2_**	59.20 (31.80, 80.20)	55.25 (36.20, 71.15)	83.30 (71.50, 83.70)	H=4.93 р=0.08
**О2•-**	10.40 (2.60, 15.10)	9.70 (3.00, 13.95)	15.50 (13.90, 16.10)	H=5.77 р=0.06
**COPD+hypertension (n=28)**				
	**M/M (n=7)**	**M/T (n=13)**	**T/T (n=8)**	
**8-isoprostane**	164.00 (138.00, 196.00)	178.00 (144.00, 189.00)	166.50 (159.00, 188.50)	H=0.01 р=1.00
**Н_2_О_2_**	82.90 (71.90, 89.60)	85.40 (73.50, 87.40)	83.50 (81.05, 83.80)	H=0.60 р=0.74
**О2•-**	15.80 (13.90, 17.30)	16.40 (14.20, 16.90)	15.60 (15.10, 16.85)	H=0.01 р=1.00
**COPD (n=25)**				
	**M/M (n=6)**	**M/T (n=18)**	**T/T (n=1)**	
**8-isoprostane**	135.50 (108.00, 162.00)	126.50 (96.00, 149.00)	169.00	H=2.72 р=0.26
**Н_2_О_2_**	55.65 (50.70, 60.70)	55.25 (46.70, 57.20)	65.80	H=2.68 р=0.26
**О2•-**	9.80 (8.90, 10.90)	9.70 (7.50, 10.60)	11.20	H=2.30 р=0.32
**Control (n=20)**				
	**M/M (n=6)**	**M/T (n=13)**	**T/T (n=1)**	
**8-isoprostane**	27.00 (26.00, 35.00)	34.00 (21.00, 41.00)	12.00	H=2.97 р=0.23
**Н_2_О_2_**	26.25 (24.70, 31.80)	31.40 (21.20, 34.10)	12.90	H=2.96 р=0.23
**О2•-**	1.50 (1.20, 2.60)	2.10 (1.00, 2.50)	0.50	H=2.77 р=0.25

Note: H = Kruskal-Wallis test; p = the significance test of the latter.

**Table 4:** Comparative analysis of changes in oxidative stress parameters in groups of COPD-only patients and patients with combined COPD and hypertension within one genotype of the AGT gene.

**Parameters**	**8-isoprostane**	**Н_2_О_2_**	**О2•-**
**M/M genotype**			
**Kruskal-Wallis test and its respective test of significance**	Н=13.25; р=0.001*	Н=16.01; р<0.001*	Н=16.04; р<0.001*
**Mann–Whitney U test in pairwise comparison of groups**	р1-2=0.074 р2-3=0.003* р1-3=0.004*	р1-2=0.003* р2-3=0.003* р1-3=0.004*	р1-2=0.003* р2-3=0.003* р1-3=0.004*
**M/T genotype**			
**Kruskal-Wallis test and its respective test of significance**	Н=32.27; р<0.001*	Н=37.68; р<0.001*	Н=37.86; р<0.001*
**Mann–Whitney U test in pairwise comparison of groups**	р1-2=0.001* р2-3<0.001* р1-3<0.001*	р1-2<0.001* р2-3<0.001* р1-3<0.001*	р1-2<0.001* р2-3<0.001* р1-3<0.001*
**T/T genotype**			
**Kruskal-Wallis test and its respective test of significance**	Н=2.58; р=0.276	Н=4.42; р=0.111	Н=4.45; р=0.108

Note: H = Kruskal-Wallis test; p = the significance test of the latter.

## Discussion

The role of ACE and AGT gene polymorphisms in the pathogenesis of comorbid COPD and hypertension is the focus of the research community’s attention. As known and as the name implies, the angiotensin-converting enzyme converts angiotensin I to angiotensin II and also inactivates the bradykinin pathway of the kallikrein–kininogen system. The main pharmacological effect of angiotensin II is vasoconstriction, which is achieved through the activation of molecules in the renin-angiotensin-aldosterone system. In addition to that, angiotensin II stimulates the synthesis of ROS and cytokines (including interleukin-6 and interleukin-8), which effectively makes it a pro-inflammatory substance [29]. ACE inhibits bradykinin (a substance producing vasodilation via enhanced formation of nitric oxide), increases the permeability of blood vessels and facilitates the synthesis of pro-inflammatory cytokines, including IL-6 and IL-8 [30]. Our previous study has not shown the different alleles of the ACE and AGT genes to have any significant influence on the incidence and course of COPD, hypertension, or a combination of the two.

Moreover, the somewhat higher incidence of the D/D genotype found in patients with comorbid COPD and hypertension (the difference was not significant) may indicate the association of this genotype with a higher risk for hypertension in subjects with COPD. In the analysis of the odds ratio, we have found a trend suggesting a protective role of the M allele of the AGT gene against COPD, hypertension, and the combination of the two. At the same time, the presence of the T allele of the AGT gene may increase the risk for the above conditions [3].

In another of our study, we have obtained data suggesting oxidative stress; in a setting of combined COPD+arterial hypertension, this factor leads to a mutual increase in the severity of both conditions [20]. Hydroperoxides (resulting from peroxidation) destroy the cellular membranes and intracellular structures due to their strong toxic effects. The disintegration of lipids in cellular membranes and the resultant massive release of fatty acids aggravates microcirculatory disorders and enhances hypoxia through activated eicosanoids formation and facilitated aggregation of blood cells, stimulation of platelet-activating factor and vasoconstriction [31]. Since cellular membranes are rich in polyunsaturated fatty acids, free radicals cause peroxidation of arachidonic acid to form isoprostanes. The biological effects of F2-isoprostanes include intense inflammation-associated activity. In this regard, the increased serum levels of 8-isoprostane found in COPD patients with or without hypertension may be suggestive of/contributory to the clinical progression of COPD [32]. In addition to the above, investigators note that cellular oxidative stress damages cell components, such as proteins, lipids, and, most importantly, DNA, which ultimately leads to mutagenesis and cell death [33,34].

Analysis of the available literature suggests the absence of studies on interrelationships between polymorphisms in the ACE and AGT genes and oxidative stress parameters in patients with comorbid COPD and hypertension. The results of our study have shown that oxidative stress parameters were not significantly dependent on the ACE and AGT gene genotypes in the group of COPD-only patients and the group of patients with comorbid COPD and hypertension. However, when test parameters of H2O2, О2•- and 8-isoprostane were compared within one genotype of the ACE gene, the most significant increase in oxidative stress parameters (compared with controls) was found in patients with COPD+hypertension and the I/I genotype. We believe these findings to be due to the lowest control values of test parameters found in subjects with the I/I genotype compared with other (I/D and D/D) ACE genotypes. The data obtained allow proposing a hypothesis of a potential protective role of the I/I genotype of the ACE gene against excessive production of free radicals. Comparable results were obtained by Tippisetty S. et al. when investigating the role of ACE polymorphism in the development of vitiligo [35]. The authors of this study believe that the I/I genotype of the ACE gene generates less ROS than other genotypes.

When comparing the test parameters of oxidative stress within one genotype of the AGT gene, the most significant increase in H2O2, О2•- and 8-isoprostane (compared with controls) was found in patients with COPD + hypertension and the M/M genotype of the AGT gene, which was also due to lower control values of test parameters in subjects with the M/M genotype compared with M/T. However, due to the small number of subjects with the T/T genotype, these data were ambiguous, which prevented us from comparing oxidative stress parameters across all genotypes of the AGT gene. As reported by population studies, the fraction of homozygous carriers of the T-allele may reach up to 10% among healthy individuals [36], which is comparable with our results obtained in the general population of subjects enrolled in the study. Moreover, it remains to be established what effects are caused by the replacement of threonine with methionine of the AGT gene in virtually healthy individuals and patients with COPD+hypertension.

## Conclusion

We did not observe any significant correlations between ACE and AGT gene polymorphisms and parameters of oxidative stress in a setting of comorbid COPD and hypertension. However, the increase in oxidative stress parameters was observed to be the most significant in patients with COPD+hypertension, and with the I/I genotype of the ACE gene, which was due to their lowest values in virtually healthy individuals. This suggests that the I/I genotype may be associated with lower levels of ROS production compared with other genotypes.

## Conflict of Interest

The authors confirm that there are no conflicts of interest.
